# Type, dose, and outcomes of physical therapy interventions for unilateral peripheral vestibular hypofunction: protocol for a systematic review

**DOI:** 10.1186/s13643-023-02328-9

**Published:** 2023-09-14

**Authors:** Carrie W. Hoppes, Eric R. Anson, Wendy J. Carender, Gregory F. Marchetti, Courtney D. Hall, Susan L. Whitney, Christiana Keinath, Susan J. Herdman

**Affiliations:** 1 Army-Baylor University Doctoral Program in Physical Therapy, Joint Base San Antonio-Fort Sam Houston, TX USA; 2 Department of Otolaryngology, University of Rochester, Rochester, NY USA; 3 Department of Otolaryngology, Michigan Medicine, University of Michigan, Ann Arbor, MI USA; 4 Department of Physical Therapy, Duquesne University, Pittsburgh, PA USA; 5 Hearing and Balance Research Program, Mountain Home VAMC, Mountain Home, TN USA; 6 Department of Rehabilitative Sciences, Physical Therapy Program, East Tennessee State University, Johnson City, TN USA; 7 Department of Physical Therapy, University of Pittsburgh, Pittsburgh, PA USA; 8 Library, Quillen College of Medicine, East Tennessee State University, Johnson City, TN USA; 9 Department of Physical Medicine and Rehabilitation, School of Medicine (Emerita), Emory University, Atlanta, GA USA

**Keywords:** Systematic review, Meta-analysis, Unilateral peripheral vestibular hypofunction, Physical therapy

## Abstract

**Background:**

Unilateral peripheral vestibular hypofunction can result in symptoms of dizziness, gaze and gait instability, and impaired navigation and spatial orientation. These impairments and activity limitations may negatively impact an individual’s quality of life, ability to perform activities of daily living, drive, and work. There is strong evidence supporting vestibular physical therapy for reducing symptoms, improving gaze and postural stability, and improving function in individuals with vestibular hypofunction. However, there is great variability in clinical practice with regard to the type of interventions and only weak evidence to guide optimal exercise dosage. It is important to identify the most appropriate interventions and exercise dosage to optimize and accelerate recovery of function and to decrease distress. The objective of this systematic review is to determine which interventions and which doses are most effective in decreasing dizziness or vertigo, improving postural control, and improving quality of life in adults with unilateral peripheral vestibular hypofunction.

**Methods:**

The literature will be systematically searched using the following online databases: PubMed/MEDLINE, EMBASE, Web of Science (Science and Social Science Citation Index), Cumulative Index for Nursing and Allied Health Literature (CINAHL), and The Cochrane Library (Cochrane Database of Systematic Reviews, Cochrane Central Register of Controlled Trials [CENTRAL], Cochrane Methodology Register). The review will include randomized controlled trials (RCTs), including cluster RCTs, to assess the beneficial effects of the interventions. Assessment of methodological quality and risk of bias will be performed by two independent, blinded reviewers using the PEDro scale and Cochrane Risk of Bias version 2, respectively. The primary outcome measure will be change in self-perceived handicap related to dizziness from baseline to the end of the study, measured using the Dizziness Handicap Inventory. Other relevant outcome measures will include self-reported change in symptoms (to include severity, frequency, and duration) such as verbal or visual analog scales for dizziness. Tertiary outcome measures will include questionnaires related to disability and/or quality of life.

**Discussion:**

This systematic review will identify, evaluate, and integrate the evidence on the effectiveness of physical therapy interventions for unilateral peripheral vestibular hypofunction in an adult population. We anticipate our findings may inform individualized treatment and future research. Clinical recommendations generated from this systematic review may inform vestibular physical therapy treatment of individuals with unilateral peripheral vestibular hypofunction.

**Trial registration:**

In accordance with the guidelines, our systematic review protocol was registered with the International Prospective Register of Systematic Reviews (PROSPERO) on 06 August 2021 (registration number CRD42021266163). In the event of protocol amendments, the date of each amendment will be accompanied by a description of the change and the rationale.

**Supplementary Information:**

The online version contains supplementary material available at 10.1186/s13643-023-02328-9.

## Background

Unilateral peripheral vestibular hypofunction is the complete or partial loss of function of one peripheral vestibular sensory organ and/or vestibulocochlear nerve [1, 2]. Acute unilateral peripheral vestibular hypofunction is most commonly due to vestibular neuritis but may also be due to trauma, surgical transection, ototoxic medications, Meniere’s disease, or other pathology of the peripheral vestibular sensory organ or vestibulocochlear nerve [1–3]. Patients with acute unilateral peripheral vestibular hypofunction present with spontaneous nystagmus and report vertigo and nausea. Visual blurring (oscillopsia), disequilibrium, fear of falling, and instability may also be present [3–5]. Symptoms are burdensome, often limiting participation in common activities of daily living including driving, work, and recreation, thereby affecting overall quality of life [6–8]. Clinically, unilateral peripheral vestibular hypofunction is diagnosed by observation of the pattern of nystagmus and a positive head impulse test [9–11]. Vestibular function testing (caloric or rotational chair for semicircular canal function; or vestibular-evoked myogenic potentials or subjective visual vertical for otolith function; or video head impulse test [vHIT] results) can also be used to aid in diagnosis [12–14].

Movement-induced error signals are required for recovery of oscillopsia, disequilibrium, and instability [10, 15–18]. Vestibular rehabilitation is an exercise-based approach used to effectively treat dizziness for individuals with unilateral peripheral vestibular hypofunction [1, 19–22]. Vestibular exercises are aimed at promoting central compensation and improving functional abilities related to vestibular loss [23]. The vestibular rehabilitation exercises are broadly categorized: (1) gaze stability (adaptation of the vestibular ocular reflex [24]); (2) postural/gait control (motor control and vestibular spinal reflex [25]); (3) habituation (desensitization to movement [26]); and (4) walking for activity [21, 27–29]. Individually prescribed exercise programs appear to have greater benefit for individuals with vestibular hypofunction [30]; and there is limited evidence that higher levels of physical activity are more beneficial than routine levels of physical activity [31]. Despite the known benefits of vestibular rehabilitation exercises, it remains unknown which of the exercise categories are necessary [32, 33] or whether a single category of exercise is sufficient for recovery after unilateral vestibular hypofunction [34].

Optimal exercise dosage information is well known for improving cardiovascular function [35, 36] and general muscle strengthening [37–40] in health and disease. In contrast, little is known about the optimal exercise dosage [41, 42], type of vestibular rehabilitation exercises that will lead to dizziness reduction [33, 34, 43], or optimal exercise progression. Together, this lack of exercise-specific knowledge may manifest as unnecessary variation in care in the treatment of peripheral vestibular hypofunction.

As a preliminary effort to address vestibular rehabilitation dosage, the updated Clinical Practice Guideline (CPG) for vestibular rehabilitation for peripheral vestibular hypofunction included some general guidance from extrapolation of existing literature. Importantly, these recommendations were based on studies that were not designed or powered to address questions of exercise type or dosage. The recommendations include gaze stabilization exercises consisting of a minimum of (1) 3 times per day for a total of at least 12 min daily for individuals with acute/subacute unilateral vestibular hypofunction; and (2) 3–5 times per day for a total of at least 20 min daily for 4–6 weeks for individuals with chronic unilateral vestibular hypofunction [44]. Based on moderate evidence, clinicians may prescribe static and dynamic balance exercises for a minimum of 20 min daily for at least 4–6 weeks for individuals with chronic unilateral vestibular hypofunction [44]. The updated CPG for vestibular rehabilitation for peripheral vestibular hypofunction did not address exercise progression. Therefore, despite the general recommendations provided in the updated CPG, whether those recommendations are optimal remains to be determined. In recognition of this, the CPG authors called for further research to examine the systematic progression of gaze stabilization and balance exercises [44].

There are currently no systematic reviews examining vestibular rehabilitation exercise dosage. Lacking a historical consensus regarding vestibular rehabilitation exercise difficulty and intensity likely contributed to difficulty synthesizing dosage based on existing studies. Recently, Klatt et al. provided a conceptual framework for vestibular rehabilitation exercise intensity and characterized the difficulty level and theoretical progression of various exercise elements of vestibular rehabilitation [45]. Others have expanded on this concept by validating intensity-rating scales for many balance tasks commonly included in vestibular rehabilitation [46, 47]. The limited work specifically examining gaze stability exercise dosage [48] has limited generalizability due to the high-tech device required for the exercises not being available for standard clinical care. It is unclear whether dosages based on a high-tech device are transferable to the typically low-tech gaze stabilization exercises.

An additional barrier to vestibular rehabilitation dosage synthesis was the common practice of global application of all domains of vestibular rehabilitation exercises for all subjects in prior studies. This “kitchen sink” approach to vestibular rehabilitation exercise prescription may explain the lack of superiority reported in three previous systematic reviews that examined the effectiveness of vestibular rehabilitation for unilateral peripheral vestibular dysfunction. Hillier and McDonnell found moderate to strong evidence that vestibular rehabilitation is safe and effective for unilateral peripheral vestibular dysfunction [20]. They found insufficient evidence to discriminate between different forms of vestibular rehabilitation exercises [20]. Similarly, Arnold et al. found that vestibular rehabilitation was effective for treating unilateral peripheral vestibular hypofunction [49]. They also found it difficult to determine superiority of one intervention over another [49]. Lilios et al. failed to find strong evidence that supervised vestibular rehabilitation was superior to unsupervised vestibular rehabilitation [50]. In contrast, Herdman et al. [51] recently found that the number of supervised therapy visits predicted meaningful improvement in gait speed. Based on the existing literature, clinicians do not have clear evidence-based guidelines for the optimal selection, prescription, or progression of exercises to treat unilateral peripheral vestibular hypofunction.

The present study differs from previous systematic reviews in that this systematic review and network meta-analysis focuses on parsing out the benefits of multiple different exercise-based interventions. In addition to the type of intervention (education; gaze stability exercise; balance exercise; gait exercise; endurance exercise; other exercise-based interventions; and habituation), the dose and progression of exercises will be explored. Dosage includes the frequency, intensity, and duration of exercise performance, along with the mode of delivery. For the latter, we will explore whether or not the exercise was supervised, unsupervised, or guided via technology (e.g., home-based using a digital video disc [DVD]). The objective of this systematic review is to determine which interventions and which doses are most effective in decreasing dizziness or vertigo, improving postural control, and improving quality of life in adults with unilateral peripheral vestibular hypofunction. Our secondary aim is to generate clinical recommendations for vestibular physical therapists treating individuals with unilateral peripheral vestibular hypofunction.

## Methods/design

The present protocol was registered within the International Prospective Register of Systematic Reviews (PROSPERO) database (registration number CRD42021266163) and follows the reporting guidance provided in the Preferred Reporting Items for Systematic Reviews and Meta-Analyses Protocols (PRISMA-P) statement [52] (see checklist in Additional file 1). The completed systematic review and meta-analysis will follow the guidance provided in the updated Preferred Reporting Items for Systematic Reviews and Meta-Analyses (PRISMA) [53] as well as the extension in the PRISMA extension for network meta-analysis [54]. Important amendments to this protocol will be updated within the PROSPERO database and documented in the full review.

### Eligibility criteria

Studies will be selected according to the criteria outlined below.

#### Study designs

We will include randomized controlled trials (RCTs), including cluster RCTs, to assess the beneficial effects of the interventions. We will exclude controlled (non-randomized) clinical trials (CCTs) or cluster trials, observational studies (including prospective and retrospective comparative cohort and case–control or nested case–control studies), cross-sectional studies, case series, and case reports. Study protocols and abstract-only records will also be excluded.

#### Participants

We will include studies that treated adult humans (18 + years old) with unilateral peripheral vestibular hypofunction. Acute and chronic, non-surgical and post-surgical cases will be included. A diagnosis of unilateral peripheral vestibular hypofunction must be supported by clinical (non-instrumented) head impulse test (HIT) or vHIT, head shaking nystagmus, caloric and/or rotary chair testing. We will exclude studies that treated other types of dizziness (those related to the cervical spine; ear, nose, and throat; central nervous system; and cardiovascular system). For example, we will exclude studies on bilateral peripheral vestibular hypofunction, benign paroxysmal positional vertigo, vestibular migraine, and concussion.

#### Interventions

We will classify interventions described in studies according to the following broad categories: education; gaze stability exercise; balance exercise; gait exercise; endurance exercise; other exercise-based interventions; and habituation. Interventions may have been used in isolation or in combination.

#### Comparators

Given the broad perspective for interventions of interest, several comparisons will be relevant to include. These may include placebo, usual care, higher versus lower intervention dosage, or different types of interventions applied with similar dosage.

#### Outcomes

The primary outcome measures will be change in self-perceived handicap related to dizziness from baseline to the end of each study, measured using the Dizziness Handicap Inventory (DHI) [55]. Secondary outcome measures will include other scales of self-reported change in symptoms (to include severity, frequency, and duration) such as verbal or visual analog scales for dizziness. Other secondary outcome measures may include the Activities-specific Balance Confidence scale, Dynamic Gait Index, Functional Gait Assessment, Timed Up and Go, gait speed, modified Clinical Test of Sensory Interaction in Balance, Sensory Organization Test, and/or Dynamic Visual Acuity. Tertiary outcome measures will include questionnaires related to disability and/or quality of life.

#### Timing

Studies will be selected for inclusion based on the length of follow-up of outcomes. Cross-sectional studies will be excluded. All studies should have a longitudinal design with both baseline and at least one follow-up measurement at least 24 h later.

#### Setting

There will be no restrictions by type of setting.

#### Language

We will include articles reported in the English language.

### Information sources

Literature search strategies will be developed using subject headings and text words related to interventions for unilateral peripheral vestibular hypofunction. The draft search strategy for PubMed/MEDLINE is presented in Additional file 2. The search terms will be adapted for use with other bibliographic databases. We will search the following electronic bibliographic databases from January 1, 1900 to December 1, 2022: PubMed/MEDLINE, EMBASE, Web of Science (Science and Social Science Citation Index), Cumulative Index for Nursing and Allied Health Literature (CINAHL), and The Cochrane Library (Cochrane Database of Systematic Reviews, Cochrane Central Register of Controlled Trials [CENTRAL], Cochrane Methodology Register). PROSPERO will be searched for ongoing or recently completed systematic reviews. We will contact study authors as needed to request missing studies.

The literature search will be restricted to studies published in the English language with human subjects. Limited resources (time and finances) preclude the inclusion of studies published in non-English languages. Studies published from January 1, 1900 until December 1, 2022 will be sought. The searches will be re-run immediately before the final analyses and further studies retrieved for inclusion. To ensure literature saturation, we will scan the reference lists of included studies or relevant reviews identified through the search.

### Study records

#### Data management

We will implement the search strategies and import all references identified into EndNote X9 (Clarivate; Philadelphia, PA). The search results from the different bibliographic databases will be combined in a single EndNote library and we will remove duplicate articles by title and/or abstract. An online technology platform (Covidence; Melbourne, Australia) will be used to manage records and data throughout the review.

#### Study selection/selection process

Titles and/or abstracts of studies retrieved using the search strategy and those from additional sources will be independently screened by two review authors to identify studies that potentially meet the inclusion criteria outlined above. The full text of these potentially eligible studies will be retrieved and independently assessed for eligibility by two review authors. Any disagreement will be resolved through discussion with a third review author.

#### Data collection process

A standardized form will be used to extract data from the included studies for assessment of study quality and evidence synthesis. Extracted information will include: study setting; study population and participant demographics and baseline characteristics; details of the intervention and comparison conditions; study methodology; study completion rates; outcomes and times of measurement; indicators of acceptability to users; information for assessment of the risk of bias. Two review authors will extract data independently, discrepancies will be identified and resolved through discussion (with a third review author where necessary). Missing data will be requested from study authors.

### Data items

Participants must be adult humans (18 + years old) with unilateral peripheral vestibular hypofunction. Acute and chronic, non-surgical, and post-surgical cases will be included. A diagnosis of unilateral peripheral vestibular hypofunction must be supported by clinical (non-instrumented) HIT or vHIT, head shaking nystagmus, caloric and/or rotary chair testing. This review will include all studies examining at least one of the following interventions: (1) education (i.e., patient education, brochures); (2) gaze stability exercise (i.e., vestibulo-ocular reflex x1 [VORx1], VORx2); (3) balance exercise (i.e., standing on firm or foam surface); (4) gait exercise (i.e., walking with head movements); (5) endurance exercise (i.e., walking, biking); (6) other exercise-based interventions (i.e., aquatic therapy); and (7) habituation (i.e., optokinetic stimuli). Comparison interventions may include placebo, usual care, higher versus lower intervention dosage, or different types of interventions applied with similar dosage.

### Outcomes and prioritization

The primary outcome measure is the DHI [55]. Secondary outcome measures will include other scales of self-reported change in symptoms (to include severity, frequency, and duration) such as verbal or visual analog scales for dizziness. Other secondary outcome measures may include the Activities-specific Balance Confidence scale, Dynamic Gait Index, Functional Gait Assessment, Timed Up and Go, gait speed, modified Clinical Test of Sensory Interaction in Balance, Sensory Organization Test, and/or Dynamic Visual Acuity. Tertiary outcome measures will include questionnaires related to disability and/or quality of life.

### Risk of bias in individual studies

Two review authors will independently assess the methodological quality and risk of bias in included studies using the PEDro scale [56] and Cochrane Risk of Bias version 2 (RoB2) [57]. The 11-item PEDro Scale assesses both the methodological quality and risk of bias of RCTs [56]. The RoB2 assesses risk of bias across 5 domains (randomization, deviation from planned analyses, missing outcomes, measurement of outcomes, and result selection) with a categorical assessment (some concerns, low, or high) of risk of bias [57]. Disagreements between the review authors over the risk of bias in studies will be resolved through discussion (with a third review author where necessary). Level I and Level II RCTs will be differentiated based on the PEDro scale score plus three additional criteria derived from the American Physical Therapy Association (APTA) Critical Appraisal Tool for Experimental Interventions (CAT-EI) [58]. Two trained reviewers will independently evaluate the quality of each RCT using the CAT-EI. Level I RCTs receive a critical appraisal score of at least 50% and include appropriate randomization, blinding, and at least 80% follow-up. Level II RCTs receive a critical appraisal score of less than 50% or the study does not meet the additional criteria of randomization, blinding, and at least 80% follow-up.

### Data synthesis

Data will be extracted from the articles accepted in the study by two members of the research team. Both members are required to agree upon the selected data for them to be included. We will group interventions into: education; gaze stability exercise; balance exercise; gait exercise; endurance exercise; other exercise-based interventions; and habituation. A narrative synthesis, conforming to the Synthesis Without Meta-analysis (SWiM) criteria [59], will be provided structured around the target population (study sample) characteristics, type of intervention(s), dosage, and type of outcome measures of the selected studies. Although clinical and research outcome measures may differ across studies, they will be grouped according to the interventions described above. Where such data are not presented in the original research article, the corresponding author will be contacted to retrieve data. Where available, *p* values of within group changes from pre- to post-test for the outcome measures will be reported in summary tables. Cohen’s *d* effect sizes from individual studies will be calculated for within group changes from pre- to post-test for the outcome measures when such data are available. Effect size will be classified as described by Cook (2008) for interpretation of the results [60] Data tables will be organized based on methodological quality and risk of bias, with higher quality studies being listed first. This quantitative analysis will provide the basis for the formal narrative synthesis.

There is not a consensus on diagnostic criteria for unilateral peripheral vestibular hypofunction and some clinical heterogeneity can be expected because of variations in a population’s characteristics and applications of interventions between studies (i.e., frequency, intensity). Despite these limitations, a meta-analysis is planned for the groups of interventions (education; gaze stability exercise; balance exercise; gait exercise; endurance exercise; other exercise-based interventions; and habituation) if they are clinically homogeneous.

### Meta-analytic approaches

#### Assessment of heterogeneity

In this meta-analysis, heterogeneity will be assessed using the *Q* statistic as a test of the null hypothesis of homogeneity. The I^2^ index will be used to estimate the degree of heterogeneity across studies. Higgins and Thompson [61] described *I*^2^ values for interpreting magnitude as percentages of 25% (*I*^2^ = 25), 50% (*I*^2^ = 50), and 75% (*I*^2^ = 75), meaning low, medium, and high heterogeneity, respectively.

#### Effect-size calculation and interpretation

Effect sizes will provide interpretable data that are independent of units of measurement and the influence of sample size. The effect sizes of changes for all primary and secondary outcomes will be estimated separately for each study at suitable testing time points from initiation of vestibular rehabilitation.

Overall effect sizes of change for studies grouped by post-intervention outcome for both intervention and control groups will be estimated as standardized mean difference (Hedges g): g = (M_fu_–Mb)/S_pooled_, where M_fu_ indicates the mean score at follow-up, M_b_ indicates the mean score at baseline (pre-intervention), and S_pooled_ indicates the estimated pooled variance across two measures with adjustment for pre- to post-intervention correlation in each outcome score.

Note that the sign of the effect size is intended to be consistent for all outcome scores (i.e., a positive effect size indicates an improvement post-intervention for any of the outcome measures). Therefore, the effect size will be calculated as (M_b_–M_fu_)/S_pooled_ for an outcome measure where improvement is a reduction in score (e.g., DHI), and (M_fu_–M_b_)/S_pooled_ for an outcome where an improvement is an increase in score (e.g., Activities-specific Balance Confidence scale).

Overall weighted effect sizes with 95% confidence intervals (CIs) for each outcome will be estimated using a random-effects model when heterogeneity is detected or with a fixed-effects model when heterogeneity is not detected. The effect sizes will be interpreted as suggested by Cohen [62]: trivial (Hedges *g* < 0.19), small (Hedges *g* = 0.20–0.49), moderate (Hedges *g* = 0.50–0.79), and large (Hedges > 0.8).

#### Mean difference between intervention and control groups

For continuous outcomes (e.g., DHI, Activities-specific Balance Confidence scale, gait speed), between-group mean difference will be calculated when outcomes were measured using the same scale, and the standardized mean difference will be used when different scales were used among different trials, with corresponding 95% CI. The standardized mean difference is defined as the between group mean outcome difference divided by the standard deviation of the outcome. If data of standard deviations are missing for statistical pooling of effect size calculation, missing standard deviations will be replaced by calculating the trial data using standard error of the mean or 95% CI.

#### Network diagram and contribution matrix

We will present a network diagram for the primary outcome (change in self-perceived handicap related to dizziness measured using the DHI), which allows visualization of the direct comparisons between the different interventions and the routine physical therapy groups. In this diagram, the nodes (circles) indicate vestibular rehabilitation interventions, and their sizes are proportional to the sample size of the respective intervention. Lines indicate direct comparisons, the width of which is proportional to the number of available studies. The contribution matrix will be used to describe the percentage contribution of each direct meta-analysis to the overall evidence. Stata software (version 17; StataCorp LLC, College Station, TX) will be used to generate the network diagram and contribution matrix.

#### Network meta-analysis

WinBUGS 1.4.3 (MRC Biostatistics Unit, University of Cambridge, UK) will be used to conduct a network meta-analysis. Given that participant characteristics (e.g., age, sex, level of disability, and time since diagnosis) could indicate violation of the transitivity assumption [63], we will summarize and compare the characteristics of the participants recruited into each trial. If the transitivity assumption is thought to be violated, we will proceed with a narrative synthesis, conforming to the SWiM criteria [59], and possibly pairwise meta-analysis. If the transitivity assumption is thought not to be violated, we will proceed with network meta-analysis. We will fit a random effects network meta-analysis model in a Bayesian framework and assume a common heterogeneity parameter across he seven types of interventions (education; gaze stability exercise; balance exercise; gait exercise; endurance exercise; other exercise-based interventions; and habituation) for the primary outcome (change in self-perceived handicap related to dizziness measured using the DHI). The random effects model assumes the variation between trials may be due to heterogeneity and not sampling variation [63, 64]. A trace plot and Brooks-Gelman-Rubin diagnostic plot [65] will be used to assess convergence. The deviance information criterion (DIC) will be used as a measure of model fit and a measure of model complexity, with a lower DIC value preferred. Assessment of the statistical heterogeneity in the entire network will be based on the magnitude of the common τ^2^ estimated from the network meta-analysis model [66, 67].

We will use the surface under the cumulative ranking curve (SUCRA) value to obtain the cumulative ranking probabilities of the best type of intervention, with a higher SUCRA value preferred. The summary of all pairwise comparisons will be presented in a league table, as well as the probabilities of ranking the type of intervention. Investigating inconsistency is not required in this network meta-analysis because there is no direct comparative evidence among the seven types of interventions.

#### Meta-regression for between-study variance

Meta-regression will be used to explore possible factors contributing to significant between-studies variance (i.e., heterogeneity). For this meta-analysis, a random-effects model will be used to test the effects of potential covariates on effect sizes. For the outcome score effect size, age, follow-up time, chronicity of condition, symptom severity, and percentage of females will be tested as covariates. Meta-regression results will be reported as beta coefficient estimates with 95% CIs, *z* scores, and *p* values for each covariate and omnibus test of model difference. The *I*^2^ will be determined to describe the total variance between studies that was explained by the meta-regression model. For the meta-regression, a post-hoc analysis will be performed to determine the power based on the number of studies included. Meta-analytic procedures will be conducted using Comprehensive Meta-Analysis (version 3.3; Biostat, Englewood, NJ) and SPSS (version 25.0; IBM Corp, Armonk, NY).

### Meta-biases

Given that studies documenting positive findings are more likely to be published than studies with negative findings, pooled results in meta-analyses can be subject to publication bias. In this meta-analysis, we will assess publication bias using visual inspection of funnel plots of the effect sizes (Hedges *g*) versus standard errors for studies grouped by time of outcome measurement. For outcomes with funnel plots indicating asymmetry as potential evidence of publication bias, we will use the Egger regression intercept test (beta coefficient, *t* value, *p* value). Publication bias will be assessed against a one-tailed Egger regression intercept test. Statistical evidence of publication bias will be further investigated with the trim-and-fill method of Duval and Tweedie [68] to estimate the number of missing studies and provide an adjusted effect size.

### Additional analyses

While subgroup analyses may be undertaken (as described above), it is not possible to specify all of the groups in advance. Supervision (supervised vs. unsupervised, in-clinic vs. home) may also be a potential subgroup to explore. If a characteristic for treating unilateral peripheral vestibular hypofunction in an adult population was overlooked in this protocol but is clearly of major importance and justified by external evidence, we will explore it and report the subgroup analyses as an unplanned post-hoc [69]. Additionally, compliance with the exercise program (adherence to the regimen, drop-out rate) and adverse effects will be explored when possible.

## Discussion

This systematic review will be performed to critically examine the literature on the treatment of unilateral peripheral vestibular hypofunction. Specifically, we will determine which vestibular rehabilitation exercises and doses are most effective in decreasing dizziness or vertigo in adults with unilateral peripheral vestibular hypofunction. Understanding which vestibular rehabilitation exercises or combinations of exercises most effectively decrease dizziness or vertigo and how/when to progress with the interventions may speed return to work and improve quality of life [44]. Additionally, this level of exercise dosing information will contribute to precision medicine applied to individualized treatment for unilateral peripheral vestibular hypofunction [70, 71] by reducing unwarranted clinical variation. Findings from this review may support the generation of specific exercise dosage and progression recommendations for physical therapists treating individuals with unilateral peripheral vestibular hypofunction. Our findings may inform revised clinical practice guidelines with stronger recommendations for optimal exercise dosage of gaze stabilization and balance exercises.

Determining the optimal exercise interventions and dosage for vestibular rehabilitation is important especially in the context of remote monitoring of vestibular rehabilitation [72, 73]. The importance of remote monitoring (telehealth) for maximizing dizziness reduction for individuals with vestibular hypofunction has been highlighted during the coronavirus disease 2019 (COVID-19) pandemic [74, 75]. Remote exercise progression may also benefit patients who have limited access to in-person vestibular rehabilitation. Van Vugt et al. compared internet-based vestibular rehabilitation to internet-based plus in-person vestibular rehabilitation with similar beneficial effects over usual care (no vestibular rehabilitation) [73]. The online training program included exercise progressions built into the software, but the optimality of those exercise progressions remains unvalidated.

There are several limitations to the scope of this review. The present review will focus only on the treatment of *unilateral* peripheral vestibular hypofunction in an adult population. A larger percentage of individuals with dizziness presenting to medical care annually have *unilateral* peripheral vestibular hypofunction [2], justifying the specificity of this review. The scope of this review is unique in that the many types of vestibular rehabilitation exercises will be modeled for individual contributions to dizziness/vertigo reduction, a novel approach in this field. This review will be limited to the English language, which may introduce the risk of publication bias. However, a recent analysis of healthcare-related systematic reviews and meta-analyses examining the inclusion of non-English language studies identified a negative statistical effect in only 9% of included studies, but those authors noted that the overall conclusions of any systematic review were not influenced by those statistical effects [76]. At the individual study level, clinical heterogeneity is expected [77, 78]. There is no consensus on diagnostic criteria for unilateral peripheral vestibular hypofunction, unlike other vestibular diseases [79–83]. The criteria used by the researchers as stated and/or referenced in each manuscript will be used for this systematic review. Differences in acuity and severity of symptoms across studies may exist [29, 84, 85], as well as differences in selected vestibular rehabilitation exercises between studies [85–88]. In some cases, vestibular rehabilitation exercises may have been developed by the same team conducting the study [24, 72, 86, 87]. We will consider this potential source of bias. Despite these limitations, this systematic review is important for identifying evidence-based interventions that decrease dizziness or vertigo in adults with unilateral peripheral vestibular hypofunction.

The results of this systematic review may inform future research in the field of vestibular rehabilitation. Clinical subtypes may exist within unilateral peripheral vestibular hypofunction [78], and identifying which interventions are most effective in decreasing dizziness or vertigo in adults with unilateral peripheral vestibular hypofunction could form the basis for RCTs exploring the timing, dosing, and progression of treatment(s). Identifying patient-related factors for responsiveness to these interventions would aid in matching the right patient to the right treatment. Clinical recommendations for physical therapists treating individuals with unilateral peripheral vestibular hypofunction arising from this review should be validated by future research.

### Supplementary Information

**Additional file 1.** PRISMA 2020 Checklist.

**Additional file 2.**

## Data Availability

The datasets used and/or analyzed during the current study are available from the corresponding author on reasonable request.

## Abbreviations

APTA: American Physical Therapy Association
CAT-EI: Critical Appraisal Tool for Experimental Interventions
CCTs: Controlled clinical trials
CENTRAL: Cochrane Central Register of Controlled Trials
CI: Confidence interval
CINAHL: Cumulative Index for Nursing and Allied Health Literature
COVID-19: Coronavirus disease 2019
CPG: Clinical Practice Guideline
DHI: Dizziness Handicap Inventory
DIC: Deviance information criterion
DVD: Digital video disc
HIT: Head Impulse Test
MeSH: Medical subject heading
PRISMA-P: Preferred Reporting Items for Systematic Reviews and Meta-Analyses Protocols
PRISMA: Preferred Reporting Items for Systematic Reviews and Meta-Analyses
PROSPERO: International Prospective Register of Systematic Reviews
RCTs: Randomized controlled trials
RoB2: Cochrane Risk of Bias version 2
SUCRA: Surface under the cumulative ranking curve
SWiM: Synthesis Without Meta-analysis
vHIT: Video Head Impulse Test
VOR: Vestibulo-ocular reflex
