# Supplementary material for: Type, dose, and outcomes of physical therapy interventions for unilateral peripheral vestibular hypofunction: protocol for a systematic review
Source: Syst Rev. 2023 Sep 14;12:164. doi: 10.1186/s13643-023-02328-9 (PMC10503155; doi:10.1186/s13643-023-02328-9)
Supplement: Supplementary file 2 — Additional file 2. [file 13643_2023_2328_MOESM2_ESM.docx]

**ADDITIONAL FILE 2**

The draft search strategy to determine which interventions and doses are most effective in decreasing dizziness or vertigo in adults with unilateral peripheral vestibular hypofunction:

("Vestibule, Labyrinth"[MeSH] OR "Vestibular Nerve"[MeSH] OR "Vestibular Nucleus, Lateral"[MeSH] OR "Vestibular Diseases"[MeSH] OR "Vestibular Neuronitis"[MeSH] OR vestibular nucleus[tiab] OR "vestibular" [tiab] OR "vestibular disorders" OR vestibulocochlear nerve[tiab] OR inner ear[tiab] OR labyrinth disease[tiab] OR vestibular disease[tiab] OR Labyrinth Vestibule[tiab] OR Vestibulum Auris[tiab] OR Ear Vestibule[tiab] OR Vestibular Apparatus[tiab] OR Saccule[tiab] OR Utricle[tiab] OR otolith[tiab] OR Visual-vestibular interaction[tiab] OR (Peripheral vestibular[tiab] AND hypofunction[tiab]) OR (Peripheral vestibular[tiab] AND loss[tiab]) OR vestibular labyrinth[tiab] OR Vestibular nervous system[tiab] OR vestibular nerve[tiab] OR Vestibular system[tiab]) AND (UNILATERAL) AND (Randomized Controlled Trial [PT] OR Randomized Controlled Trial [MH] OR Randomized Controlled Trial [tiab] OR Controlled Trial [tiab] OR Clinical trial [tiab]) AND ("Rehabilitation"[Mesh] OR Rehabilitation[tiab] OR exercise[tiab] OR exercises[tiab] OR "Physical Stimulation"[MeSH] OR "Exercise Therapy"[ MeSH] OR "Exercise Movement Techniques"[ MeSH]) NOT "Benign Paroxysmal Positional Vertigo"[ MeSH]
